# Hospital Regulations and the Insurance Act

**Published:** 1913-01-18

**Authors:** Thos. Alex. Cook

**Affiliations:** Chairman of the General Committee of Management of the West Ham and Eastern General Hospital.


					Hospital Regulations and the Insurance Act.
To the Editor of The Hospital.
Sir,?It may interest you to know that last night at the
committee meeting of the West Ham and Eastern General
Hospital we had a long discussion with regard to the work
of the hospital under the National Insurance Act after
ihe 15th inst., and unanimously adopted St. Bartholo-
mew's Hospital rules.?Yours faithfully,
Thos. Alex. Cook,
Chairman of the General Committee of
Management of the West Ham and
Eastern General Hospital.
?January 9, 1913.

				

